# Nature-Inspired Approach: A Novel Rat Optimization Algorithm for Global Optimization

**DOI:** 10.3390/biomimetics9120732

**Published:** 2024-12-01

**Authors:** Pianpian Yan, Jinzhong Zhang, Tan Zhang

**Affiliations:** School of Electrical and Photoelectronic Engineering, West Anhui University, Lu’an 237012, China; 15357187791@163.com (P.Y.); zhangjinzhongz@126.com (J.Z.)

**Keywords:** rat optimization algorithm (ROA), Levy flight strategy, benchmark test functions, swarm-intelligence, engineering optimization issues

## Abstract

This work presents a rat optimization algorithm (ROA), which simulates the social behavior of rats and is a new nature-inspired optimization technique. The ROA consists of three operators that simulate rats searching for prey, chasing and fighting prey, and jumping and hunting prey to deal with optimization issues. The Levy flight strategy is introduced into the ROA to keep the algorithm from running into issues with slow convergence and local optimums. The ROA is tested with four real-world engineering optimization issues and twenty-two benchmark functions. Experiments show that the ROA is particularly effective at solving real-world optimization problems compared to other well-known optimization techniques.

## 1. Introduction

Stochastic optimization approaches have been used to handle a number of complex issues related to practical engineering optimization. As a combination of a stochastic algorithm and local search algorithm, the meta-heuristic algorithm has been widely used because of its practicability, strong stability, good flexibility, and high operation efficiency, such as in shop scheduling, path planning, parameter optimization, and so on. The optimization mechanism of the meta-heuristic algorithm does not rely too much on the structural information of the issue to be solved and can be applied to many types of combinatorial optimization or function optimization. The basic idea of the meta-heuristic algorithm is based on evolution and genetics, and information exchange and survival of the fittest are carried out through reproduction, variation, and competition among individuals in a group so as to approach the optimal solution step by step. Recently, scholars have developed outstanding meta-heuristic algorithms such as the water wave optimization (WWO) [1], the sine cosine algorithm (SCA) [2], the dragonfly algorithm (DA) [3], the cuckoo search (CS) [4], the ant line optimizer (ALO) [5], and the artificial bee colony (ABC) [6] to resolve optimization issues. There are various categories into which the meta-heuristic algorithms are divided, including those based on swarm intelligence, physics, and evolution [7].

The phenomenon of physics serves as inspiration for physics-based algorithms. Physics laws concerning the inertia force, material heating and cooling, the law of universal gravitation, electromagnetic field theory, and others are employed to describe these phenomena. Classical physics-based optimization algorithms include the gravitational search algorithm (GSA) [8], the galaxy-based search algorithm (GbSA) [9], central force optimization (CFO) [10], the ray optimization algorithm (RO) [11], the black hole algorithm (BH) [12], and others. Another important type of meta-heuristic algorithm is evolutionary-based algorithms, which are generated by simulating the regularity of biological evolution on earth; for example, selection, recombination, mutation, reproduction, etc. Evolutionary algorithms are devised by the researchers to seek out the optimal values for the issue to be optimized through iterative optimization searches. The advantages of algorithms include the capacity to deal with complex nonlinear issues, the requirement of an initial solution that is not high, and the ability to search for optimal values in parallel. They are especially suitable for optimization issues that are difficult to resolve with traditional methods, such as complex combinatorial optimization, coefficient optimization in machine learning, and so on. The well-known evolutionary algorithms include the biogeography-based optimizer (BBO) [13], the evolution strategy (ES) [14], genetic algorithms (GAs) [15], and differential evolution (DE) [16].

The swarm intelligence algorithm is a computational method that simulates the behavior of natural organisms to solve optimization issues. These algorithms are often enlightened by crowd behaviors in nature, such as ants foraging for food or birds flying in flocks, and seek optimal values for problems by modeling these behaviors. The advantages of a swarm intelligence optimization algorithm mainly comprise high efficiency and strong robustness, which are especially suitable for large-scale and high-dimension optimization issues. The classical swarm intelligence algorithms include particle swarm optimization (PSO), which simulates the social activity of animals such as birds and fish and seeks the extreme value in the search space through cooperation and competition among individuals. This algorithm is simple and easy to implement, and the parameter setting is flexible, so it is widely used in various optimal-value-seeking issues [17]. In recent years, many effective swarm intelligence algorithms have been proposed, such as the whale optimization algorithm (WOA), enlightened by the hunting behavior of humpback whales [18], and the wild horse optimizer (WHO), which mimics the way wild horses socialize [19].

Rat swarm optimization (RSO) is a relatively new optimization technique that has been proposed in the last three years by simulating rat foraging behavior [7]. By dynamically altering parameters, this simple and easy-to-implement algorithm seeks to achieve a good balance between exploration and exploitation. To verify the effectiveness of the RSO approach, its performance is compared with eight other popular algorithms. After that, it is used on six constrained engineering design challenges from practical applications. The technology of node localization in wireless sensor networks was presented in [20] using the RSO. In [21], the RSO is utilized to determine the optimum settings for the AlexNet architecture’s hyperparameters in order to maximize COVID-19 diagnosis accuracy. To attain the ideal PV cell and module parameters, ref. [22] introduced the RSO as a dependable, strong, and precise optimization method. In order to guarantee stability, a novel technique based on the RSO for regulating and monitoring the fluctuation characteristics was constructed in [23] to improve the reliability of loading frequency control in electrical systems. Unfortunately, the original RSO has limitations, including the following: (I) it tends to fall into local solutions; (II) its exploitation is lower than its exploration capacity; and (III) its convergence speed is slow.

To improve these shortcomings, researchers have proposed some improvement strategies, such as the RSO hybrid optimization algorithm based on the Levy flight strategy [24]. Following the Levy distribution, the Levy movement is an arbitrary searching path that alternates between short and irregularly long walks. But, incorporating Levy flight cannot fundamentally balance the local and global search of the RSO algorithm. In [25,26], the opposition-based learning strategy is introduced into RSO to enhance the performance of searching for the maximum and minimum solutions. As is well known, reverse learning strategies increase population diversity by generating reverse populations. This method can improve the global search capability of the algorithm and avoid getting stuck in local optima, but it cannot further improve the local search ability of the algorithm. The original RSO mainly resolves the optimization issue through two processes: chasing prey and fighting with prey. In order to model the complex behaviors of rats during foraging, such as the random search for prey, chasing, jumping, and rolling around prey, we need to enrich the RSO to better balance exploration and exploitation processes and achieve faster convergence speed and higher computational accuracy. In order to enhance the robustness of the RSO in complex optimization problems, the parameter *A* in chasing the prey is changed to achieve better exploration and exploitation over the iterations [27]. In [27], merely modifying the stage of chasing prey is not enough to describe the social behavior of rats, and it is difficult to obtain an excellent local search ability. The worst solution with the highest fitness value (minimum value problem) will be replaced by a new solution to improve the RSO’s local and global search capabilities and address the issue of search agents sometimes becoming stuck in the local optimum and not changing during the follow-up process [28]. The novel method swaps out a least-ranked rat’s position with its opposite, increasing the population diversity and enhancing global search ability. However, the local search performance and convergence speed of the improved RSO have not been substantially changed in theory. Similarly, in [29], the solution vector, or randomly produced position, is enclosed in the population of the RSO to increase population diversity and the global search ability. In order to avoid the system falling into local optima and achieve higher optimization accuracy, [30] combines the particle swarm optimization and rat search algorithm to enhance the efficiency of the RSO. We are aware that the alternating use of algorithms in a hybrid optimization algorithm may cause algorithm instability during the iteration process. Additionally, the hybrid algorithm’s computational complexity will rise, and its convergence speed will be somewhat impacted. In [31], the rat swarm optimization algorithm has been truly improved, taking into account the social behaviors of rats, including jumping, rotating, attacking, escaping, chasing, etc., in the process of algorithm improvement. However, [31] does not model rat social behavior mathematically but only modifies the parameter *A* in the chasing prey phase and provides the working principle of the jump operator. Although the addition of these strategies to RSO can improve the convergence speed and calculation accuracy, it is still much inferior to some excellent swarm intelligence algorithms such as WOA and WHO. Considering the shortcomings in the improvement of RSO mentioned above, this paper conducts detailed mathematical modeling of the social behaviors of rats, including the random search for prey, chasing, jumping, and rolling around prey, to create a new rat optimization algorithm (ROA). The novel aspects of the ROA include: (I) in the initial stage of algorithm execution, that is, when parameter A≥2.5, rats randomly search for prey to improve the global search ability; (II) the best and random search agents are chosen for updating the location of the search agents when A≥2.5 and A≥2.5, respectively, to increase the population diversity; (III) the random number q, evenly distributed between [0,1], is introduced into the algorithm. According to the size of q, the ROA performs a switch between jumping in a semi-circular form around prey and chasing and fighting with prey, enhancing the local search ability; and (IV) to expand the search space and avoid the algorithm falling into the local optima, the Levy motion strategy is introduced into the ROA originally proposed in this paper. The addition of the Levy flight strategy is not due to the author’s lack of trust in the performance optimization ability of the presented algorithm but rather an attempt to further improve it.

The study consists of the following sections: Section 2 provides a detailed introduction to the mathematical modeling process of the rat optimization algorithm proposed for the first time in this article. Section 3 shows the simulation results on the 22 benchmark test functions. In Section 4, the optimization performance of the proposed rat optimization algorithm with and without the Levy flight strategy is tested on four practical engineering design issues and compared with some well-known algorithms. Section 5 provides the conclusion and some future work directions at the end.

## 2. Rat Optimization Algorithm (ROA)

### 2.1. Inspiration

Rats are generally socially intelligent by nature. They engage in a range of social activities, including chasing and fighting with prey, jumping and rolling in a semi-circular form around the prey, etc. [7]. This study’s primary source of inspiration is the semi-circular jumping around the prey, chasing, fighting, and attacking activities of rat groups during predation. To create a new rat optimization algorithm (ROA) for optimization, these actions throughout the rat predation process are described mathematically. It should be noted that the proposed ROA is clearly divided into two phases: (i) the exploration phase: the rats randomly search for prey, chase, and fight with prey; and (ii) the exploitation phase: the rats jump in a semi-circular form around prey and attack the prey.

### 2.2. Chasing and Fighting with Prey (Including the Exploration Phase)

Rats have a keen sense of smell and the ability to find food, discover danger, and identify fellows. In addition, rats search for targets by screening rather than by olfactory tracking. When rats search for prey within a limited, familiar area, the screening method is more effective than the tracking method. This means that the sense of smell is an essential instinct for rats when searching for food, pursuing mates, or evading pursuit. Although rats also rely on other senses such as hearing and vision, smell plays a central role in their search for food and detection of danger. After discovering prey, rats in a rat colony will transmit information through specific behaviors. This behavior involves using one’s own behavior or bodily signs to act on the sensory organs of other rats, thereby altering their behavior. Rats can detect the location of prey and chase and fight with it through the shortest distance (similar to linear pursuit). Since the optimal location is unknown in the whole search space, the ROA algorithm assumes that the current optimal solution is the prey location or the rat location closest to the prey. Other search agents will then update their location based on the set best search agent to be closer to the best search agent. This hunting behavior of rats can be expressed by the following formula:

(1) $$D=A\cdot X\left(t\right)+C\cdot \left(X^{*}\left(t\right)-X\left(t\right)\right)$$

(2) $$X\left(t+1\right)=\left|X^{*}\left(t\right)-D\right|$$

where D describes the location update, $X^{*}\left(t\right)$ stands for the best ideal location sought thus far, and X(t) expresses the locations of rats. The parameters A and C are expressed by the following equations:

(3) $$\begin{array}{c} A=R-t\times \left(\frac{R}{Inter_{\max}}\right)\\ C=2\cdot rand \end{array}$$

where $t=0,1,2,\ldots ,Inter_{\max}$ with $Inter_{\max}$ denoting the maximum number of iterations, and R and C are randomly located in the intervals [1,5] and [0,2], respectively. Parameter A is also used to regulate the exploration and exploitation phase in the iterative process. According to (3), we know that A is a random parameter in [0,5] and the fluctuation range of the parameter A decreases with the increase in iteration times.

During the hunting process, rats spend a significant amount of time searching for prey. Therefore, we have included an exploration phase in the algorithm proposed in this article. The division between the exploration phase and the chase phase is based on the value of parameter A. Accordingly, in comparison with (1) and (2), a randomly selected search agent is employed for location updates throughout the search for prey rather than the ideal search agent that has been calculated up to now. This approach forces exploration and permits a global search to be carried out by the ROA algorithm when A≥2.5. This behavior is modeled as follows:

(4) $$D=A\cdot X\left(t\right)+C\cdot \left(X_{rand}\left(t\right)-X\left(t\right)\right)$$

(5) $$X\left(t+1\right)=\left|X_{rand}\left(t\right)-D\right|$$

where $X_{rand}\left(t\right)$ is a randomly selected rat (a random location) from the current population.

### 2.3. Jumping in a Semi-Circular Form Around Prey and Attacking Prey (Including the Exploitation Phase)

When faced with prey, rats will adopt the strategy of containment action and use teamwork to capture prey. This behavior demonstrates teamwork and hunting intelligence. For example, when the rats find prey, they will quickly gather together, forming a circle around the prey to ensure a successful capture. In addition, while hunting prey, each rat faces the prey and makes a random jump in a semi-circular form. This behavior is modeled as follows:

(6) $$D=C\cdot \left(X^{*}\left(t\right)-X\left(t\right)\right)$$

(7) $$X\left(t+1\right)=X^{*}\left(t\right)-D\cdot \Xi^{r}\sin\left(\frac{\pi \iota }{4}\right)$$

where Ξ>1 is a positive constant and r=(a−1)⋅rand+1 with $a=-1-\frac{t}{Inter_{\max}}$. The parameters Ξ and r are employed to adjust the distance between the semi-circular (rat position) and the prey.

We refer to the above location update method as semi-circular jump updating; that is, this strategy first obtains the distance between the rat and prey using (6). Then, a semi-circular jump Equation (7) is established when the rat faces the prey.

It is important to note that rats can chase and fight their prey in a straight line and can approach their prey in a semi-circular arc simultaneously. To simulate this simultaneous behavior, we assume that during optimization there is a 50% chance of choosing to chase and fight with prey or jump around prey and hunt prey to update the rat location. The mathematical model is as follows:

(8) $$X\left(t+1\right)=\left\{ \begin{array}{ll} \left|X^{*}\left(t\right)-D\right|, & q<0.5\\ X^{*}\left(t\right)-D\cdot \Xi^{r}\sin\left(\frac{\pi \iota }{4}\right), & q\geq 0.5 \end{array}\right.$$

where q is randomly located in the interval [0,1].

At the beginning of the ROA algorithm, we take a random population as the initial solution. In the process of constant iteration, search agents update their locations based on the best result from the previous iteration or a randomly selected search agent. The fluctuation interval of the A parameter is reduced from 5 to 0 as the number of iterations increases, and this parameter is used to adjust the balance of the exploration and exploitation. The best and random search agents are chosen for updating the location of the search agents when A<2.5 and A≥2.5, respectively. According to the size of q, the ROA performs a switch between either jumping in a semi-circular form around prey and attacking prey or chasing and fighting with prey. Finally, the ROA is aborted by satisfying the termination condition. In conclusion, because it includes the exploration and exploitation phases, the ROA can be viewed as a global optimizer. It should be noted that there are mainly two parameters in the ROA that need to be adjusted, that is, A and C.

**Figure 1:**
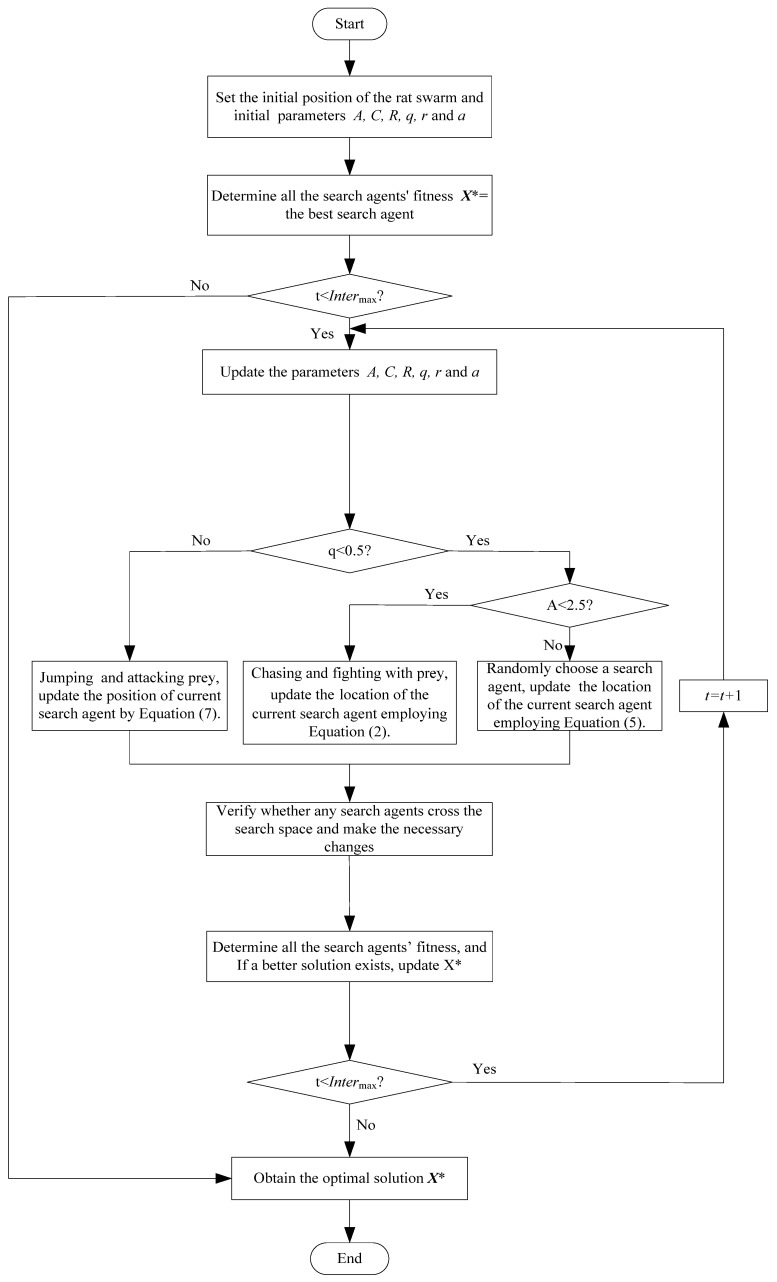
Flow chart of the ROA (* denotes the best ideal location sought thus far).

Algorithm 1 displays the ROA’s pseudocode and a flowchart of the ROA is provided in Figure 1.

**Algorithm 1** Rat optimization algorithm without Levy flight strategy**Begin** **Step 1.** Set the initial position of the rat swarm $X_{i}(i=1,2,\ldots ,n)$ **Step 2.** Determine all the search agents’ fitness     $X^{*}$ = the best search agent **Step 3. while** ($t<Inter_{\max}$) do       **for** each search agent Update A, C, R, q, *r*, and a         **if 1** (q<0.5) **if 2** (A<2.5) Update the location of the current search agent employing Equation (2) **else if 2** (A≥2.5) Randomly choose a search agent $X_{rand}$ Update the location of the current search agent employing Equation (5) **end if 2**         **else if 1** (q≥0.5) Update the location of the current search agent employing Equation (7) **end if 1** **end for**       Verify whether any search agents cross the search space and make the necessary changes       Determine all the search agents’ fitness If a better solution exists, update $X^{*}$ t=t+1     **end while**     return $X^{*}$ **End**

The proposed ROA mainly includes parameters *A*, *C*, *R*, *q*, *r*, and *a*. Similar to the original RSO, parameters R and C are randomly distributed in intervals [1, 5] and [0, 2], respectively. According to (3), it can be inferred that the parameter *A* is randomly located within the interval [0, 5], and the fluctuation range decreases with the increase in iteration times. In the RSO, the parameters *A* and *C* are used to achieve good exploitation and exploration. In the proposed ROA, the parameter *A* is given a different capability, i.e., the best and random search agents are chosen for updating the location of the search agents when A<2.5 and A≥2.5, respectively, to achieve a better balance between exploitation and exploration. Rats may simultaneously approach their prey in a semi-circular arc or chase and fight them in a straight line in the ROA. In order to model this simultaneous behavior, we assume that, in order to update the rat’s location during optimization, there is a 50% chance of selecting to jump around prey and hunt prey or chase and fight with prey. Therefore, the parameter *q* in (8), which is randomly located in the interval [0, 1], can achieve random switching between two hunting modes. As the number of iterations increases, the parameter *a* decreases linearly from −1 to −2. Considering the relationship between parameters *r* and *a*, it can be inferred that r is randomly distributed in the interval [−2, 1]. The purpose of selecting parameter r in this way is to randomly adjust the distance between the rat and the prey.

### 2.4. Further Improvement of the ROA

We know that any excellent swarm intelligence optimization algorithm can be further improved after being proposed. Similarly, to expand the search space and avoid the algorithm falling into the local optima, the Levy motion strategy is introduced into the ROA originally proposed in this paper. The addition of the Levy flight strategy is not due to the author’s lack of trust in the performance optimization ability of the presented algorithm but rather an attempt to further improve it. In the following content, we will give a comparison experiment between the ROA algorithm and its improved algorithm, from which readers can see that the optimization ability of the two algorithms is roughly the same.

Using the Levy flight strategy, the location update is expressed as follows [32]:

(9) $$x_{ij}\left(t+1\right)=x_{ij}\left(t\right)+usign\left(rand-0.5\right)\times Levy$$

where i denotes the ith individuals and j indicates the number of individuals, u expresses a random number with uniform distribution, and sign(⋅) is a sign function.

Obedient to the Levy distribution is the relationship between the step length of the Levy flight and time t. The location is computed as follows for the probability density function of the Levy flight strategy:

(10) $$Levy∼u=t^{-del},\;\;\;\;\;\;\;\;\;1<del\leq 3$$

where del denotes a coefficient of power. The generated random step length of the Levy flight strategy is computed using Mantegna’s algorithm. Here’s how the location is computed:

(11) $$epsilon=\frac{w}{{\left|z\right|}^{1/1.5}}\;\;\;\;\;\;\;w∼N(0,Sigma_{w}^{2})\;\;\;\;\;\;\;z∼N(0,Sigma_{z}^{2})$$

where epsilon denotes a random step length, and the normal distributions are obeyed by w and z. $Sigma_{w}$ and $Sigma_{z}$ are computed in the following ways, respectively:

(12) $$Sigma_{w}={\left[\frac{2.5Gamma\cdot \sin(0.75\pi )}{1.875Gamma2^{0.25}}\right]}^{1/1.5}\;\;\;\;\;\;\;Sigma_{z}=1$$

where Gamma expresses the standard gamma function.

**Figure 2:**
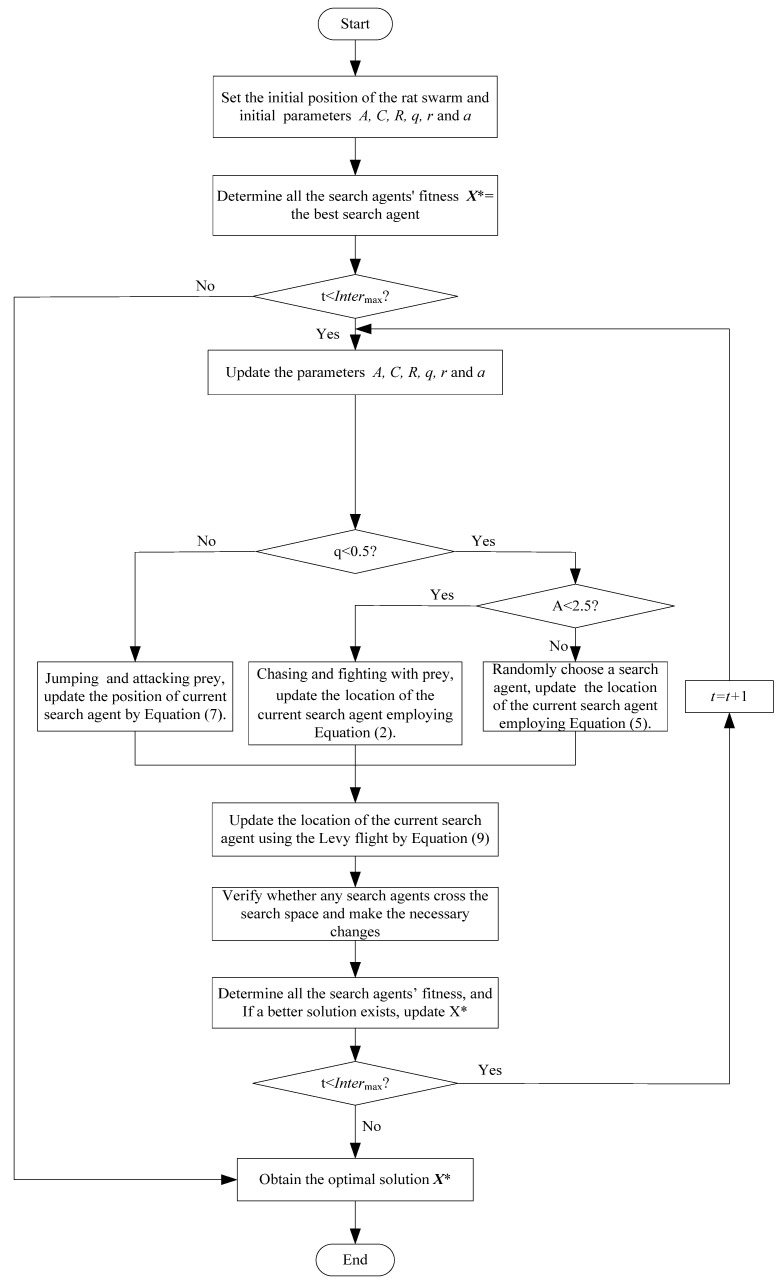
Flow chart of the ROA with the Levy flight strategy.

The further improved ROA (IROA) is named the im-algorithm, and the only difference between Algorithms 1 and im-algorithm is the addition of the Levy flight strategy in the IROA. Therefore, the pseudocode of the ROA with the Levy flight strategy will not be given here. Nevertheless, we give the flow chart of the ROA with the Levy flight strategy in Figure 2 to help readers better comprehend the ROA proposed for the first time in this study as well as the ROA with the Levy flight strategy.

### 2.5. Time Complexity and Space Complexity

The time complexity of the ROA proposed in this study for the first time is analyzed in this subsection. The ROA without the Levy flight strategy primarily consists of four steps, namely initialization, chasing and fighting with prey (including the exploration phase), jumping in a semi-circular form around prey and attacking prey (including the exploitation phase), and halting judgment. n, d, and $Inter_{\max}$ are employed to express the population size, define the dimension of the issue to be optimized, and express the upper limit of iteration numbers in simulation, respectively. A double loop (n and d times) is included in the initialization, and the corresponding time complexity can be expressed as O(n∗d) operations. Each operation in chasing and fighting with prey as well as jumping in a semi-circular form around prey and attacking prey contains a triple loop (n, $Inter_{\max}$, and d times), and the corresponding time complexity includes $O\left(n*Inter_{\max}*d\right)$ operations. Because the time complexity of the halting judgment is O(1), the total time complexity of the ROA without the Levy flight strategy is $O\left(n*Inter_{\max}*d\right)$. The amount of computer storage space required to run the algorithm is described by the space complexity of the ROA algorithm. Feasible solutions are used to estimate the space complexity of the ROA without the Levy flight strategy. Therefore, the total space complexity (the maximum amount of space that will be used at any given time, as computed upon initialization) of the ROA is O(n∗d). It can be seen that the time and space complexity of the ROA with the Levy flight strategy is equal to those of the ROA without the Levy flight strategy because of the time complexity of updating positions using the Levy flight strategy (there is a triple loop: n, $Inter_{\max}$, and d times) is $O\left(n*Inter_{\max}*d\right)$, and the computer storage space occupied by the Levy flight strategy overlaps with the space occupied by the ROA algorithm.

## 3. Experimental Results and Discussion

In order to illustrate how well the presented ROA and the ROA with the Levy flight strategy perform, this section carries out experiments on 22 benchmark test functions. Three categories are involved in the benchmark functions. As listed in Table 1, $f_{1}∼f_{7}$ are unimodal, $f_{8}∼f_{13}$ are multimodal, and $f_{14}∼f_{22}$ are fixed-dimension multimodal.

To illustrate the performance of the proposed ROA and the ROA with the Levy flight strategy, the simulation of the seven optimization algorithms is carried out in this section. These metaheuristic optimization algorithms include the rat swarm optimizer (RSO) [7], RSO with the Levy flight strategy (LRSO) [24], particle swarm optimization (PSO) [17], the wild horse optimizer (WHO) [19], the whale optimization algorithm (WOA) [18], the rat optimization algorithm (ROA), and the improved ROA with the Levy flight strategy (IROA). The parameter settings of all algorithms are provided in Table 2. The simulations of all the algorithms are performed in the Matlab R2022b version that is installed on Microsoft Windows 11 with a Core i7 processor with 2.60 GHz and 16 GB of memory. For the swarm intelligence algorithm involved in this paper, the corresponding population size, number of iterations, and number of independent runs are all set as 50, 1000, and 20, respectively. The optimum value, worst value, mean value, and standard deviation are expressed by Best, Worst, Mean, and Std.

Benchmark functions are a series of functions used to test and evaluate the performance of optimization algorithms. These functions generally include various types of functions, such as unimodal functions, multimodal functions, fixed-dimension multimodal functions, etc., to simulate the complexity and difficulty of various optimization problems in the real world. In addition, each benchmark test function has a well-defined search space and a known global or approximate optimal solution. By running optimization algorithms on these functions and comparing them with known optimal solutions, the performance of optimization algorithms can be effectively evaluated. Unimodal test functions $f_{1}∼f_{7}$, as their names suggest, have a single optimum, allowing them to evaluate an algorithm’s exploitation and convergence. The unimodal benchmark test function reflects many optimization problems in real-world engineering, such as resource allocation, process optimization, network design, etc. Optimization algorithms improve efficiency and accuracy by finding the optimal solution. On the other hand, multimodal and fixed-dimension multimodal test functions $f_{8}∼f_{22}$ are more challenging than unimodal functions since they have several optimal solutions. While the others are referred to as local optima, one is referred to as the global optimum. To find the global optimum, an algorithm should steer clear of all local optima. Thus, the multimodal and fixed-dimension multimodal test functions can be used to test algorithms for the exploration and avoidance of local optima. The multimodal benchmark test function reflects many practical engineering problems involving multiple local optimal solutions, such as mechanical design optimization, aircraft trajectory optimization in aerospace design, economic scheduling in power system optimization, and finding new materials with specific properties in materials science and engineering. However, due to the complexity of multimodal optimization problems, finding the global optimal solution remains challenging.

From Table 3, we can see that both rat optimization algorithms with and without the Levy flight strategy can seek out the ideal minimum value of the functions $f_{1}$, $f_{2}$, $f_{3}$, and $f_{4}$. Especially, the optimal, worst, mean, and standard deviation values of the functions $f_{1}$, $f_{2}$, $f_{3}$, and $f_{4}$ sought out by the rat optimization algorithm with the Levy flight strategy are all 0, pointing out that the proposed rat optimization algorithm has the capacity to acquire the global optimal solution. The best and mean values of the function $f_{5}$ sought out by the rat optimization algorithm with the Levy flight strategy and the rat optimization algorithm proposed in this paper are clearly superior to those of the algorithms being compared. In addition, the comparison of simulation findings between the rat optimization algorithm and the rat optimization algorithm with the Levy flight strategy for $f_{5}$ also shows that the addition of different strategies may not further improve the original algorithm. For $f_{6}$, the whale optimization algorithm performs the best, followed by the proposed rat optimization algorithm with the Levy flight strategy and the rat optimization algorithm. A careful comparison of simulation results reveals that the rat optimization algorithm and the rat optimization algorithm with the Levy flight strategy have similar optimization effects to the whale optimization algorithm. However, the search for optimal results of the rat optimization algorithm and rat optimization algorithm with the Levy flight strategy presented in this study is better than those of the rat swarm optimizer with or without the Levy flight strategy. For $f_{7}$, the best, worst, and standard deviation values of the rat optimization algorithm with and without the Levy flight strategy are superior to those of the rat swarm optimizer with and without the Levy flight strategy, the particle swarm optimization, the wild horse optimizer, and the whale optimization algorithm; however, the mean value of the rat optimization algorithm is worse than that of the rat swarm optimizer with the Levy flight strategy. This also shows that the Levy flight strategy can increase population diversity to avoid premature entry into local optimization. The simulation findings for unimodal functions represent that the devised rat optimization algorithm with the Levy flight strategy has a certain degree of robustness, stability, speedy convergence, and better computational accuracy.

In Table 4, for function $f_{8}$ with a minimum value of −12569, although the optimal value sought out by the whale optimization algorithm is best, the rat optimization algorithm with the Levy flight strategy comes in second place, and the best value obtained is about the same as the whale optimization algorithm. Furthermore, both the rat optimization algorithm with the Levy flight strategy and the rat optimization algorithm’s worst, mean, and standard deviation values are superior to those of the other comparison approaches, demonstrating that the rat optimization algorithm can successfully resolve the function optimization issue by avoiding too-early convergence. For functions $f_{9}$, $f_{10}$, and $f_{11}$, using the rat optimization algorithm with and without the Levy flight strategy, the rat swarm optimizer with and without the Levy flight strategy, the wild horse optimizer, and the whale optimization algorithm can find the optimal solution. However, the worst, mean, and standard deviation values found by the rat optimization algorithm with and without the Levy flight strategy and the rat swarm optimizer with the Levy flight strategy tie for first place and are superior to those of the other algorithms. The best values of the functions $f_{12}$ and $f_{13}$ can be sought out by the wild horse optimizer and particle swarm optimization, respectively. But the best values searched by the rat optimization algorithm with and without the Levy flight strategy are better than those sought out by the rat swarm optimizer with and without the Levy flight strategy, respectively. Therefore, the simulation outcomes state clearly that the proposed rat optimization algorithm has a great improvement compared with the traditional rat swarm optimizer.

It can be seen from Table 5 that the proposed rat optimization algorithm with the Levy flight strategy can seek out the global optimal solution of the function $f_{14}$, which is second only to the optimal value searched by the wild horse optimizer. Even so, the worst, mean, and standard deviation values sought out by our proposed rat optimization algorithm are still the best among all algorithms. For the function $f_{15}$, the optimal value sought out by the rat optimization algorithm is the best among all algorithms. For the functions $f_{16}$ and $f_{21}$, all algorithms can accurately seek out the optimal value. However, the worst, mean, and standard deviation values sought out by the whale algorithm and particle swarm optimization are not equal to the optimal value. From the simulation outcomes of the functions $f_{17}$, $f_{18}$, and $f_{19}$, it can be observed that the particle swarm optimization and wild horse optimizer seek out the minimum values for the functions $f_{17}$ and $f_{19}$ that are smallest, and the whale optimization algorithm seeks out the minimum value for the function $f_{18}$ that is the most ideal. However, the worst, mean, and standard deviation values for the functions $f_{17}$, $f_{18}$, and $f_{19}$ sought out by the rat optimization algorithm are the best among all algorithms. It can be seen from the worst solution, mean solution, and standard deviation that the rat optimization algorithm with the Levy flight strategy has stronger global search ability, higher convergence accuracy and computational efficiency, and better stability and robustness. For $f_{20}$, the rat optimization algorithm without the Levy flight strategy seeks out the exact solution −1, but the worst solution, mean solution, and standard deviation sought out by it are worse than those obtained by the particle swarm optimization and wild horse optimizer. The optimal solution and standard deviation sought out by all of the original rat swarm optimizers with or without the Levy flight strategy and the proposed rat optimization algorithm with or without the Levy flight strategy are the minimum values of the function $f_{22}$. But only the worst solution and mean solution sought out by the proposed rat optimization algorithm with the Levy flight strategy are equal to the optimal value. Overall, based on the simulation findings of all test functions, it can be seen that the devised rat optimization algorithm with the Levy flight strategy has strong global search ability, higher convergence accuracy and computational efficiency, and higher stability and robustness. In addition, the presented rat optimization algorithm with and without the Levy flight strategy has improved the optimization performance of the original rat swarm optimizer with and without the Levy flight strategy, respectively, to a certain extent, especially in the optimization process of fixed-dimension multimodal functions.

**Figure 3:**
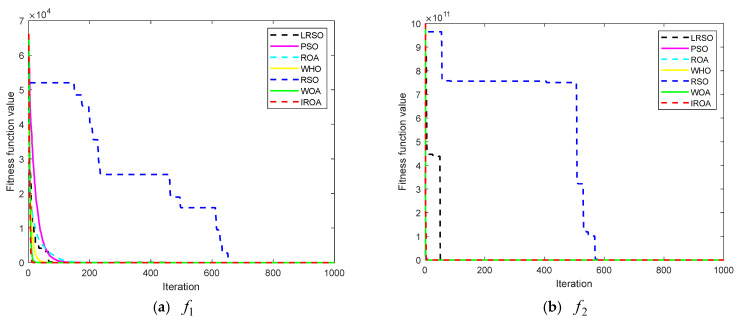

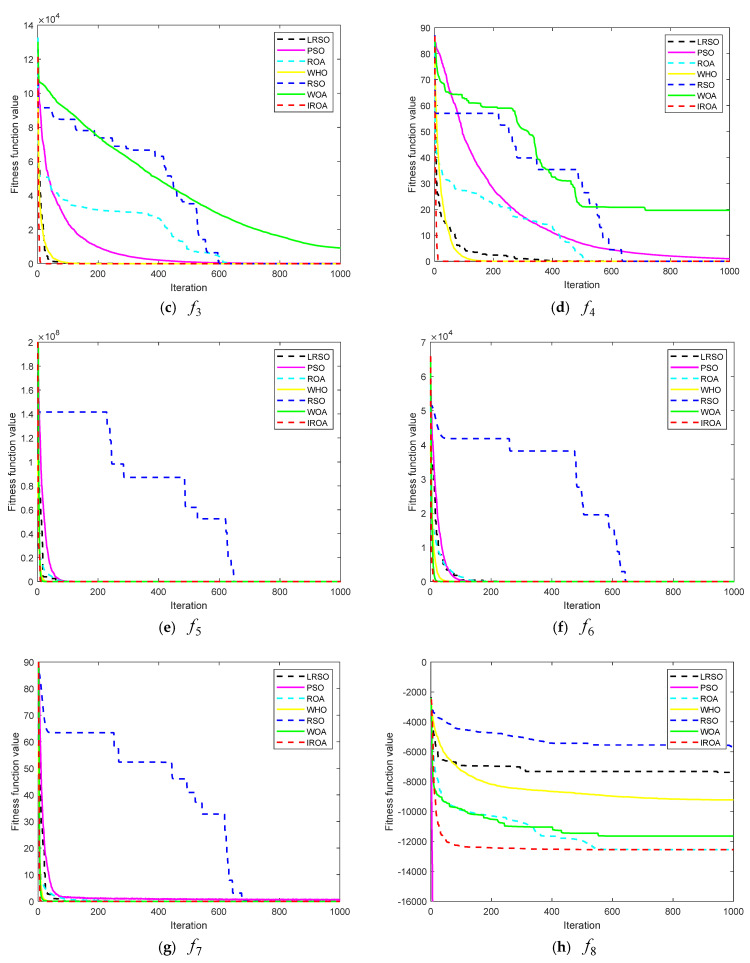

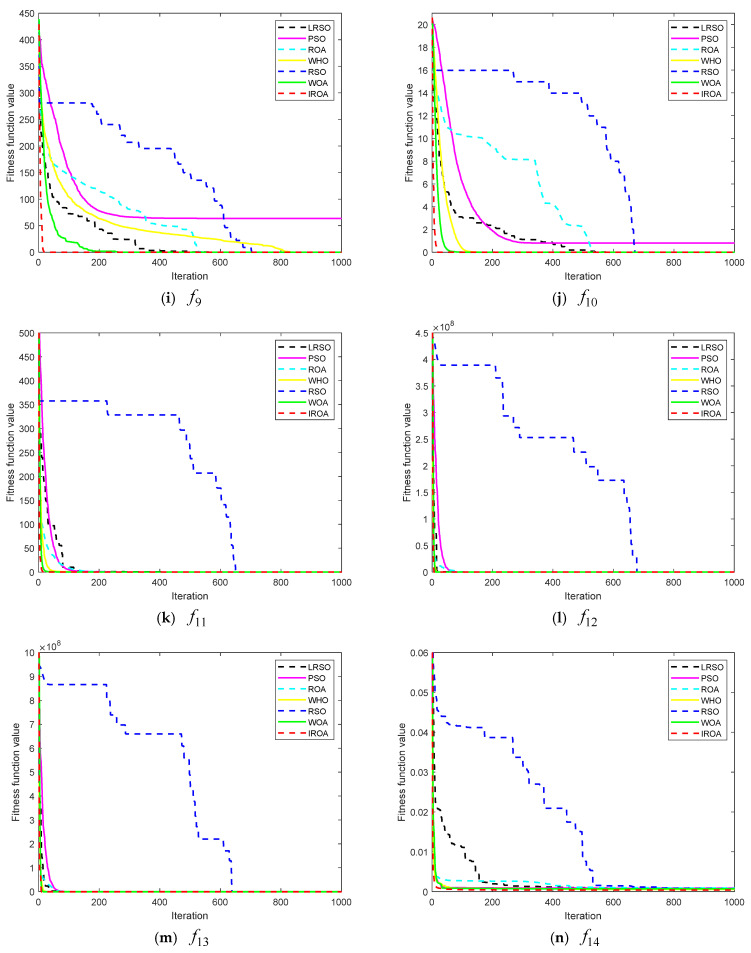

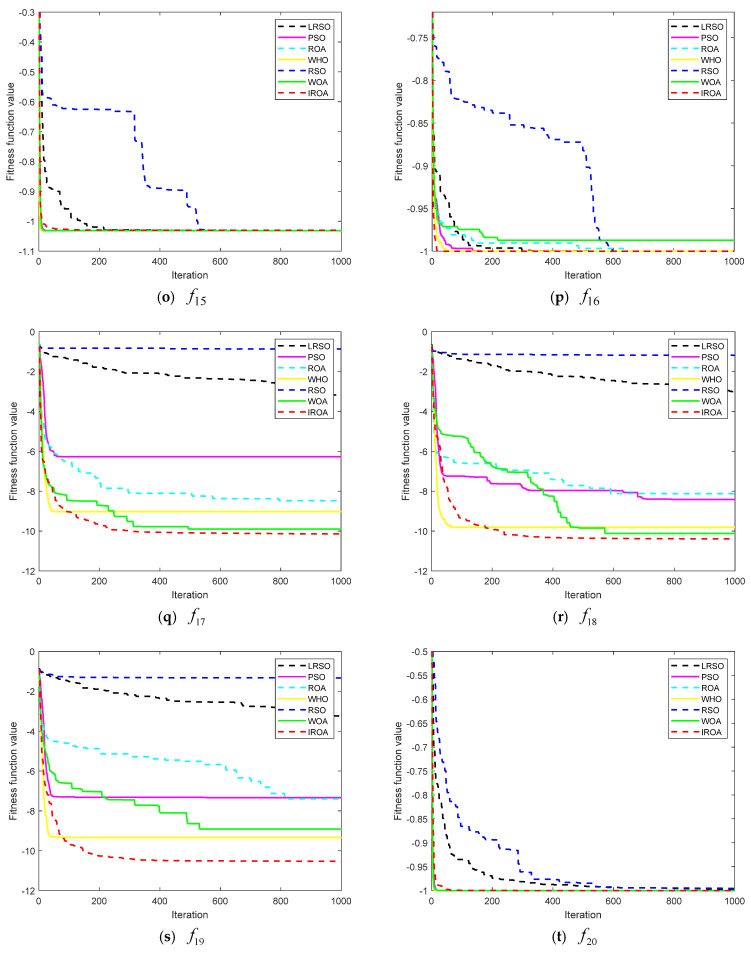

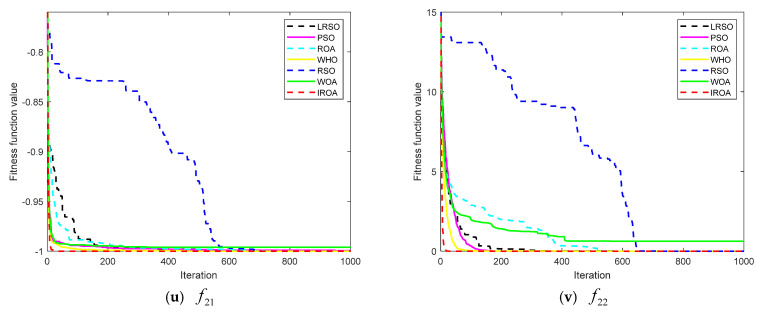
Convergence curve of these algorithms when solving the benchmark functions.

The convergence curve of all the algorithms involved in this article used to resolve the function optimization issue is shown in Figure 3. The test function’s convergence curve is an intuitive display of the algorithm’s optimization accuracy and rate of convergence. It is simple to demonstrate the advantages of the suggested rat optimization algorithm’s optimization performance by contrasting the convergence curves regarding all algorithms. From the convergence curves regarding the unimodal functions $f_{1}-f_{7}$, it can be observed that the convergence speed of the proposed rat optimization algorithm without the Levy flight strategy is significantly faster than that of the rat swarm optimizer without the Levy flight strategy. The convergence speed of the rat optimization algorithm with the Levy flight strategy is clearly increased as contrasted to the rat swarm optimizer with or without the Levy flight strategy. Moreover, the optimization accuracy and convergence speed of the rat optimization algorithm with or without the Levy flight strategy have been improved to a certain extent as contrasted to other algorithms, as shown in Table 3 and Figure 3. From (h–m) in Figure 3, especially (h–j), it can be seen that the rat optimization algorithm with the Levy flight strategy increases the diversity of the population and makes it easier to escape from local optima. Therefore, the proposed algorithm has stronger global search ability and can find better optimal solutions. For $f_{14}-f_{22}$, the rat optimization algorithm with the Levy flight strategy can basically seek out the minimum values, and in addition, the optimization accuracy of the rat optimization algorithm with the Levy flight strategy is significantly higher than that of the rat swarm optimizer. Moreover, it can be seen from the convergence curves regarding all algorithms that the proposed algorithm has the best overall computational accuracy and convergence speed. Further analysis of the simulation findings shows that the convergence speed and computational accuracy of both the rat swarm optimizer and the rat optimization algorithm with the Levy flight strategy are higher than those of both the rat swarm optimizer and the rat optimization algorithm without the Levy flight strategy, which also shows that the Levy flight strategy can make the original algorithm jump out of the local optima to seek out a better solution. Overall, the rat optimization algorithm with or without the Levy flight strategy, which is proposed in this paper for the first time, is a practical and efficient approach for handling function optimization problems.

**Figure 4:**
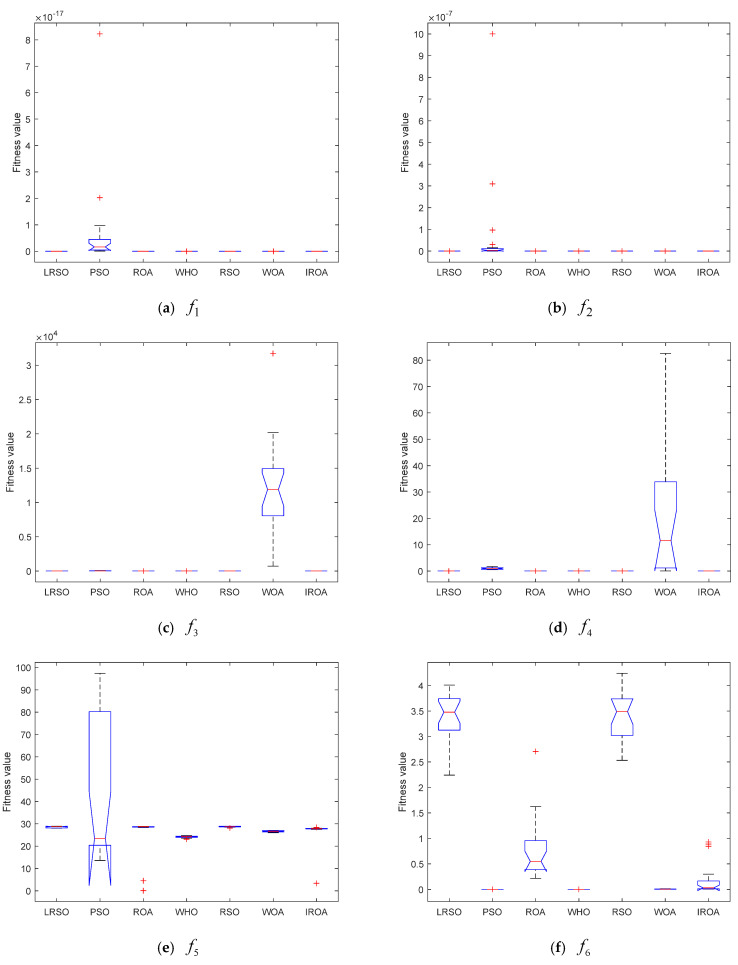

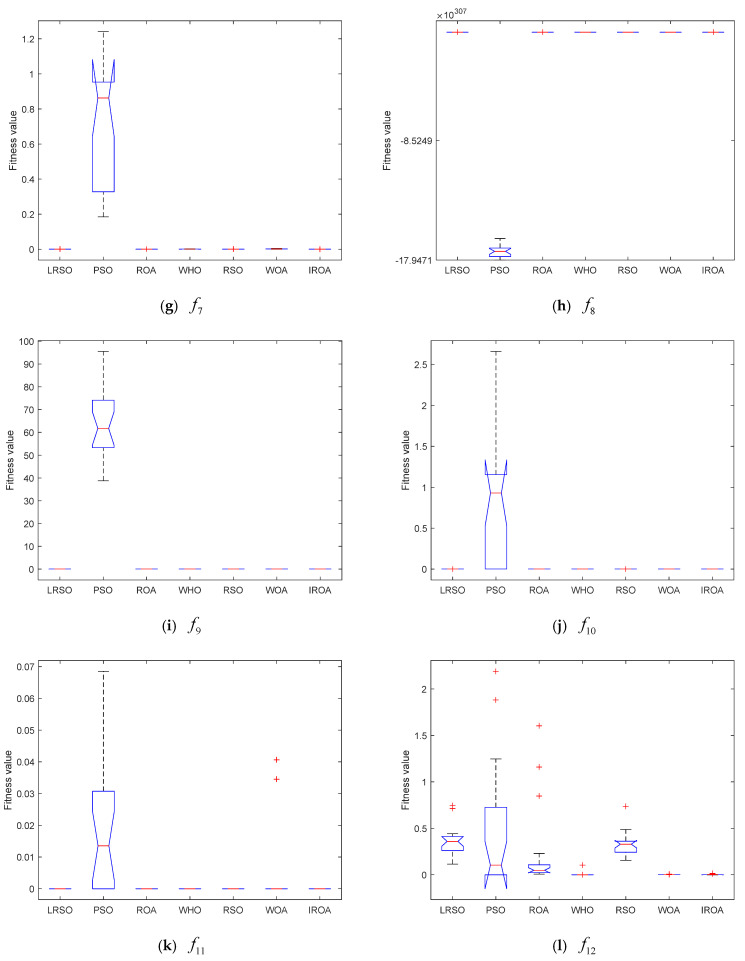

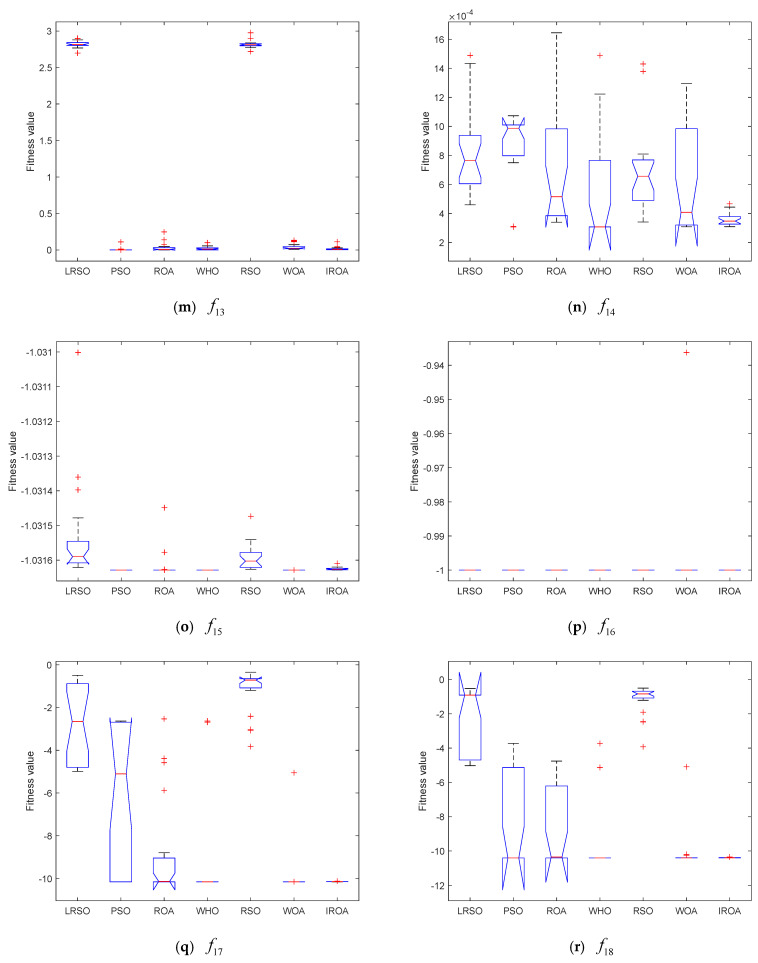

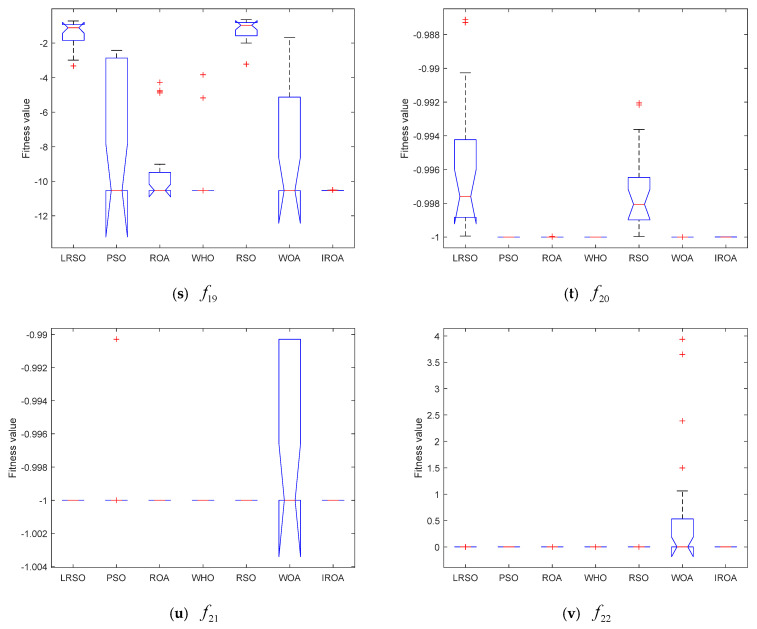
ANOVA tests of these algorithms when solving the benchmark functions.

The ANOVA test findings for the algorithm involved in this paper to resolve the function optimization issue are displayed in Figure 4. We know that ANOVA analysis can obtain quantitative indicators about the stability of the algorithm so as to conduct a more comprehensive evaluation of the performance of the algorithm. In addition, ANOVA is very useful for evaluating the performance of the optimization algorithm under different environments or conditions and thus showing the stability of the optimization algorithm. For the unimodal functions $f_{1}-f_{7}$, except for the particle swarm optimization, which has a relatively large standard deviation, the standard deviations of all other algorithms are roughly the same. After careful observation, it can be seen that the standard deviation of the devised rat optimization algorithm is slightly smaller than that of the rat swarm optimizer algorithm, showing that the presented algorithm has better optimization performance. For the multimodal functions $f_{8}-f_{13}$, the standard deviations of the rat optimization algorithm with the Levy flight strategy are overall better than those of the other algorithms. It should be noted that the standard deviations of the rat swarm optimizer are significantly larger than those of the rat optimization algorithm, which also means that the rat optimization algorithm has stronger global and local search capabilities to seek out more accurate optimal values. For the fixed-dimension multimodal functions $f_{14}-f_{22}$, the magnitude of the standard deviations of the proposed rat optimization algorithm with the Levy flight strategy has obvious advantages over other algorithms. The standard deviations of the rat swarm optimizer are greater than those of the rat optimization algorithm. The optimization performance and convergence speed of the rat optimization algorithm perform better than those of the rat swarm optimizer. This shows that the rat optimization algorithm has stronger robustness and stability.

Assume that n, d, and $Inter_{\max}$ express the population size, the dimension of the issue to be optimized, and the upper limit of iteration numbers in simulation, respectively. The time complexity of the algorithms mainly used for experiments in this section is summarized as follows. In [7], the time complexity of the RSO is $O\left(n*Inter_{\max}*d\right)$. The time complexity of the PSO is $O\left(n*Inter_{\max}*d\right)$ [17]. After viewing the relevant literature [18,32] on the WOA algorithm, it is found that the time complexity of WOA is also $O\left(n*Inter_{\max}*d\right)$. The overall computational complexity of WHO is $O\left(2*n*Inter_{\max}*d+n*d+Nfoal*Inter_{\max}\right)$ [33]. The ROA primarily consists of four steps, namely initialization, chasing and fighting with prey (including the exploration phase), jumping in a semi-circular form around prey and attacking prey (including the exploitation phase), and halting judgment. A double loop (n and d times) is included in the initialization, and the corresponding time complexity can be expressed as O(n∗d) operations. Each operation in chasing and fighting with prey as well as jumping in a semi-circular form around prey and attacking prey contains a triple loop (n, $Inter_{\max}$, and d times), and the corresponding time complexity includes $O\left(n*Inter_{\max}*d\right)$ operations. Because the time complexity of the halting judgment is O(1), the total time complexity of the ROA is $O\left(n*Inter_{\max}*d\right)$. Since the time complexity of updating positions using the Levy flight strategy (there is a triple loop: n, $Inter_{\max}$, and d times) is $O\left(n*Inter_{\max}*d\right)$, the time complexity of the LRSO in [24] and IROA is $O\left(n*Inter_{\max}*d\right)$ too. It should be noted that the mathematical models of the ROA and WOA are similar, so the computational complexity of the two algorithms is the same.

## 4. ROA for Engineering Design Issues

The paper’s involved algorithms are applied to resolve the following engineering design issues in order to confirm the viability and efficacy of the rat optimization algorithms with and without the Levy flight strategy: the piston lever design issue [34,35], welded beam design issue [36,37,38], gear train design [39,40,41], and car side impact design issue [42,43].

### 4.1. Piston Lever Design Issue

**Figure 5:**
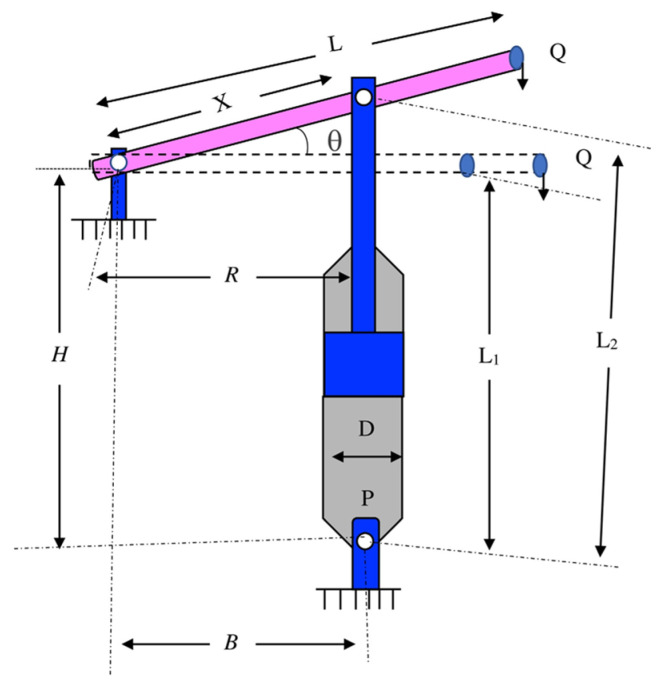
Piston lever design issue.

As seen (displayed) in Figure 5, the primary goal of this optimization issue is to locate the piston components and minimize the oil volume when the piston lever is pulled up from $0^{{}^{\circ}}$ to $45^{{}^{\circ}}$. Four decision variables, H, B, X and D, are included in this optimization issue. The mathematical description of the optimization design is given as follows:

Consider

(13) $$\chi =[\chi_{1}\;\;\;\chi_{2}\;\;\;\chi_{3}\;\;\;\chi_{4}]=[H\;\;\;B\;\;\;D\;\;\;X]$$

Minimize

(14) $$f(\chi )=\frac{1}{4}\pi \chi_{3}^{2}(L_{2}-L_{1})$$

Subject to

(15) $$G_{1}(\chi )=QL\cos\theta -RF\leq 0$$

(16) $$G_{2}(\chi )=Q(L-\chi_{4})-M_{\max}\leq 0$$

(17) $$G_{3}(\chi )=\frac{6}{5}\times (L_{2}-L_{1})-L_{1}\leq 0$$

(18) $$G_{4}(\chi )=\frac{\chi_{3}}{2}-\chi_{2}\leq 0$$

(19) $$R=\frac{\left|-\chi_{4}(\chi_{4}\sin\theta +\chi_{1})+\chi_{1}(\chi_{2}-\chi_{4}\cos\theta )\right|}{\sqrt{{\left(\chi_{4}-\chi_{2}\right)}^{2}+\chi_{1}^{2}}}$$

(20) $$F=\frac{\pi P\chi_{3}^{2}}{4}$$

(21) $$L_{1}=\sqrt{{\left(\chi_{4}-\chi_{2}\right)}^{2}+\chi_{1}^{2}}$$

(22) $$L_{2}=\sqrt{{\left(\chi_{4}\sin\theta +\chi_{1}\right)}^{2}+{\left(\chi_{2}-\chi_{4}\cos\theta \right)}^{2}}$$

(23) $$\theta =45^{{}^{\circ}},\;\;\;Q=10000lbs,\;\;\;L=240in,\;\;\;M_{\max}=1.8\times 10^{6}lbs\;\;in,\;\;\;P=1500psi$$

Variable range

(24) $$0.05\leq \chi_{1},\chi_{2},\chi_{4}\leq 500,\;\;\;\;\;\;0.05\leq \chi_{3}\leq 120$$

It can be seen from the findings in Table 6 that the rat optimization algorithm with the Levy flight strategy, in contrast to other algorithms, can seek out the smallest cost in the piston lever design issue. Furthermore, the rat swarm optimizer with the Levy flight strategy can seek out the second-smallest cost regarding the piston lever design. This also shows that the Levy flight strategy has indeed enhanced the global search capability of these two algorithms. On the whole, the rat optimization algorithm with the Levy flight strategy obtains the global optimal solution with greater stability and robustness, which lowers the cost of the piston lever design issue and enhances the algorithm’s optimization efficiency.

### 4.2. Welded Beam Design Issue

**Figure 6:**
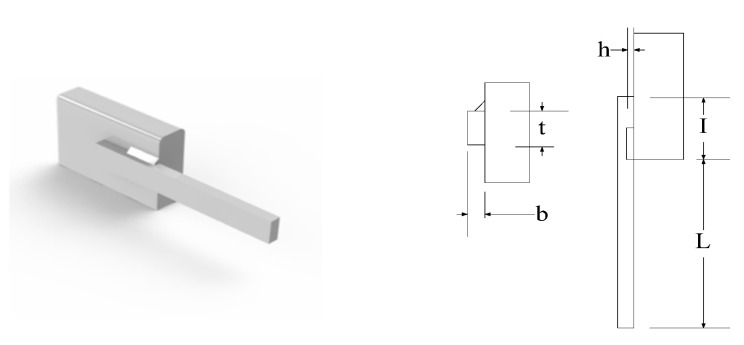
Structure of the welded beam.

The welded beam design issue, as a typical structural engineering design, aims to achieve the minimum production cost. The relevant decision parameters, as shown in Figure 6, are provided as follows: bar thickness (b), bar height (h), clamped bar length (l), and weld thickness (h). The mathematical description of the optimization design is provided as follows:

Consider

(25) $$\chi =[\chi_{1}\;\;\;\chi_{2}\;\;\;\chi_{3}\;\;\;\chi_{4}]=[h\;\;\;l\;\;\;t\;\;\;b]$$

Minimize

(26) $$f(\chi )=1.10471\chi_{1}^{2}\chi_{2}+0.04811\chi_{3}\chi_{4}(14.0+\chi_{2})$$

Subject to

(27) $$\begin{array}{l} g_{1}(\chi )=\tau (\chi )-\tau_{\max}\leq 0\\ g_{2}(\chi )=\sigma (\chi )-\sigma_{\max}\leq 0\\ g_{3}(\chi )=\delta (\chi )-\delta_{\max}\leq 0\\ g_{4}(\chi )=\chi_{3}-\chi_{4}\leq 0\\ g_{5}(\chi )=P-P_{c}(\chi )\leq 0\\ g_{6}(\chi )=0.125-\chi_{1}\leq 0\\ g_{7}(\chi )=1.1047\chi_{1}^{2}+0.04811\chi_{3}\chi_{4}(14.0+\chi_{2})-5.0\leq 0 \end{array}$$

where $0.1\leq \chi_{1}\leq 2$, $0.1\leq \chi_{2}\leq 10$, $0.1\leq \chi_{3}\leq 10$, $0.1\leq \chi_{4}\leq 2$, $\tau (\chi )=\sqrt{{\left(\tau^{\prime }\right)}^{2}+2\tau^{\prime }\tau^{\prime \prime }\frac{\chi_{2}}{2R}+{\left(\tau^{\prime \prime }\right)}^{2}}$, $\tau^{\prime }=\frac{P}{\sqrt{2}\chi_{\chi }\chi_{2}}$, $\tau^{\prime \prime }=\frac{MP}{J}$, $M=P(L+\frac{X_{2}}{2})$, $R=\sqrt{\frac{\chi_{2}^{2}}{4}+{\left(\frac{\chi_{1}+\chi_{3}}{2}\right)}^{2}}$, $J=2\left\{\sqrt{2}\chi_{1}\chi_{2}\left[\frac{\chi_{2}^{2}}{4}+{\left(\frac{\chi_{1}+\chi_{3}}{2}\right)}^{2}\right]\right\}$, $\sigma (\chi )=\frac{6PL}{\chi_{4}\chi_{3}^{2}}$, $\delta (\chi )=\frac{6PL}{E\chi_{3}^{2}\chi_{4}}$, and $P_{c}(\chi )=\frac{4.103E\sqrt{\frac{\chi_{3}^{2}\chi_{4}^{6}}{36}}}{L^{2}}\left(1-\frac{\chi_{3}}{2L}\sqrt{\frac{E}{4G}}\right)$.

We can observe from the outcomes in Table 7 that the rat optimization algorithm without the Levy flight strategy, compared to other algorithms, can seek out the smallest optimal cost in the welded beam design issue. Then, the rat optimization algorithm with the Levy flight strategy can obtain the second-smallest optimal cost regarding the welded beam design. The simulation findings demonstrate that the rat optimization algorithm seeks out the global optimal solution with greater convergence accuracy, which decreases the design cost and strikes a good balance between exploration and exploitation.

### 4.3. Gear Train Design Issue

**Figure 7:**
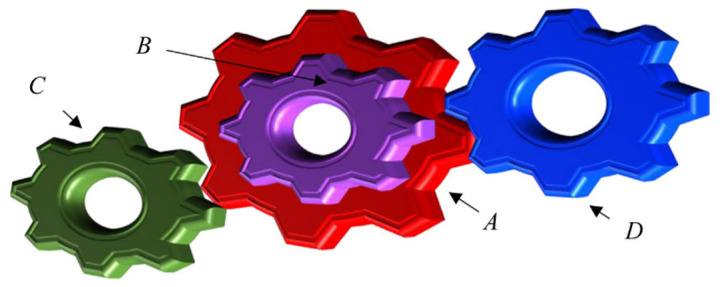
Gear train design issue.

This design issue, as shown in Figure 7, aims to seek out the best teeth number and the minimum cost of the gear ratio. The relevant decision parameters include the number of gear teeth $N_{A}$, $N_{B}$, $N_{C}$ and $N_{D}$. By using mathematical tools, the design requirement is stated as follows:

Consider

(28) $$\chi =[\chi_{1}\;\;\;\chi_{2}\;\;\;\chi_{3}\;\;\;\chi_{4}]=[N_{A}\;\;\;N_{B}\;\;\;N_{C}\;\;\;N_{D}]$$

Minimize

(29) $$f(\chi )={\left(\frac{1}{6.931}-\frac{\chi_{3}\chi_{2}}{\chi_{1}\chi_{4}}\right)}^{2}$$

Variable range

(30) $$12\leq \chi_{i}\leq 60,\;\;\;i=1,2,\ldots ,4$$

All of the algorithms’ optimization findings regarding the gear train design issue are shown in Table 8. We can see that the rat optimization and whale optimization algorithms mainly involved in this work can seek out the lowest manufacturing cost regarding the gear train design. In addition, the corresponding design parameters sought out by the two algorithms are good enough. The findings show that the rat optimization algorithm possesses better optimization accuracy when searching the global optimal value and great feasibility for handling the gear train design issue.

### 4.4. Car Side Impact Design Issue

**Figure 8:**
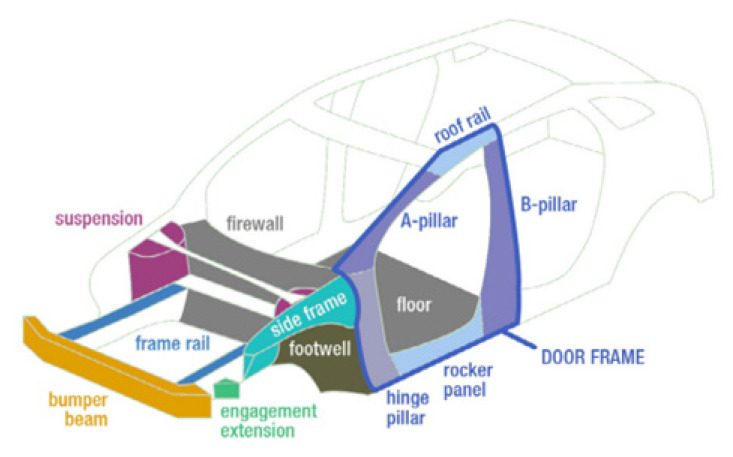
Car side impact design issue.

This optimized manufacturing design, as depicted in Figure 8, aims to seek out the minimum weight of the car by fully considering the car design variables. The relevant design parameters are mainly composed of the B-pillar’s inner thickness ($\chi_{1}$), the B-pillar reinforcement’s thickness ($\chi_{2}$), the floor side inner thickness ($\chi_{3}$), the cross members’ thickness ($\chi_{4}$), the door beam’s thickness ($\chi_{5}$), the door beltline reinforcement’s thickness ($\chi_{6}$), the roof rail’s thickness ($\chi_{7}$), the materials’ thickness of the B-pillar inner ($\chi_{8}$), the floor side inner thickness ($\chi_{9}$), the barrier height ($\chi_{10}$), and the hitting position ($\chi_{11}$). The mathematical description of this optimization issue is provided as follows:

Consider

(31) $$\chi =[\chi_{1}\;\;\;\chi_{2}\;\;\;\chi_{3}\;\;\;\chi_{4}\;\;\;\chi_{5}\;\;\;\chi_{6}\;\;\;\chi_{7}\;\;\;\chi_{8}\;\;\;\chi_{9}\;\;\;\chi_{10}\;\;\;\chi_{11}]$$

Minimize

(32) $$f(\chi )=1.98+4.90\chi_{1}+6.67\chi_{2}+6.98\chi_{3}+4.01\chi_{4}+1.78\chi_{5}+2.73\chi_{7}$$

Subject to

(33) $$\begin{array}{ll} g_{1}(\chi )= & 1.16-0.3717\chi_{2}\chi_{4}-0.00931\chi_{2}\chi_{10}-0.484\chi_{3}\chi_{9}\\ & +0.01343\chi_{6}\chi_{10}\leq 1 \end{array}$$

(34) $$\begin{array}{l} g_{1}(\chi )=1.16-0.3717\chi_{2}\chi_{4}-0.00931\chi_{2}\chi_{10}-0.484\chi_{3}\chi_{9}\\ \;\;\;\;\;\;\;\;\;\;\;\;\;\;+0.01343\chi_{6}\chi_{10}\leq 1 \end{array}$$

(35) $$\begin{array}{l} g_{3}(\chi )=0.214+0.00817\chi_{5}-0.131\chi_{1}\chi_{8}-0.0704\chi_{1}\chi_{9}\\ \;\;\;\;\;\;\;\;\;\;\;\;\;\;+0.03099\chi_{2}\chi_{6}-0.018\chi_{2}\chi_{7}+0.0208\chi_{3}\chi_{8}+0.121\chi_{3}\chi_{9}\\ \;\;\;\;\;\;\;\;\;\;\;\;\;\;-0.00364\chi_{5}\chi_{6}+0.0007715\chi_{5}\chi_{10}-0.000535\chi_{6}\chi_{10}\\ \;\;\;\;\;\;\;\;\;\;\;\;\;\;+0.00121\chi_{8}\chi_{11}\leq 0.32 \end{array}$$

(36) $$\begin{array}{l} g_{4}(\chi )=0.074-0.061\chi_{2}-0.163\chi_{3}\chi_{8}+0.001232\chi_{3}\chi_{10}\\ \;\;\;\;\;\;\;\;\;\;\;\;\;\;-0.166\chi_{7}\chi_{9}+0.227\chi_{2}^{2}\leq 0.32 \end{array}$$

(37) $$\begin{array}{l} g_{5}(\chi )=28.98+3.818\chi_{3}-4.2\chi_{1}\chi_{2}+0.0207\chi_{5}\chi_{10}+6.63\chi_{6}\chi_{9}\\ \;\;\;\;\;\;\;\;\;\;\;\;\;\;-7.7\chi_{7}\chi_{8}+0.32\chi_{9}\chi_{10}\leq 32 \end{array}$$

(38) $$\begin{array}{l} g_{6}(\chi )=33.86+2.95\chi_{3}+0.1792\chi_{10}-5.057\chi_{1}\chi_{2}-11.0\chi_{2}\chi_{8}\\ \;\;\;\;\;\;\;\;\;\;\;\;\;\;-0.0215\chi_{5}\chi_{10}-9.98\chi_{7}\chi_{8}+22.0\chi_{8}\chi_{9}\leq 32 \end{array}$$

(39) $$g_{7}(\chi )=46.36-9.9\chi_{2}-12.9\chi_{1}\chi_{8}+0.1107\chi_{3}\chi_{10}\leq 32$$

(40) $$\begin{array}{l} g_{8}(\chi )=4.72-0.5\chi_{4}-0.19\chi_{2}\chi_{3}-0.0122\chi_{4}\chi_{10}\\ \;\;\;\;\;\;\;\;\;\;\;\;\;\;+0.009325\chi_{6}\chi_{10}+0.000191\chi_{11}^{2}\leq 4 \end{array}$$

(41) $$\begin{array}{l} g_{9}(\chi )=10.58-0.674\chi_{1}\chi_{2}-1.95\chi_{2}\chi_{8}+0.02054\chi_{3}\chi_{10}\\ \;\;\;\;\;\;\;\;\;\;\;\;\;\;-0.0198\chi_{4}\chi_{10}+0.028\chi_{6}\chi_{10}\leq 9.9 \end{array}$$

(42) $$\begin{array}{l} g_{10}(\chi )=16.45-0.489\chi_{3}\chi_{7}-0.843\chi_{5}\chi_{6}+0.0432\chi_{9}\chi_{10}\\ \;\;\;\;\;\;\;\;\;\;\;\;\;\;-0.0556\chi_{9}\chi_{11}-0.000786\chi_{11}^{2}\leq 15.7 \end{array}$$

Variable range

(43) $$0.5\leq \chi_{1}-\chi_{7}\leq 1.5,\;\;\;\;\;\;\chi_{8},\chi_{9}\in [0,1],\;\;\;\;\;\;-30\leq \chi_{10},\chi_{11}\leq 30$$

It can be seen from the simulation findings in Table 9 that the optimal cost of the rat optimization algorithm without or with the Levy flight strategy has been reduced significantly in contrast to those of the compared algorithms in the car side impact design issue. Among all algorithms, the rat optimization algorithm, without or with the Levy flight strategy, can seek out the smallest car weight. The simulation findings demonstrate that the rat optimization algorithms, without or with the Levy flight strategy, effectively balance exploration and exploitation, enhance optimization ability, and possess stronger stability and higher optimization accuracy in resolving the car side impact design.

## 5. Conclusions and Future Research

This study proposes a novel swarm-intelligence-based optimization algorithm going by the name of the rat optimization algorithm (ROA) for the first time. To further enhance the global search ability, the rat optimization algorithm is combined with the Levy flight strategy and called the improved rat optimization algorithm (IROA). To verify the optimization performance, the rat optimization algorithm with and without the Levy flight strategy is used to solve the optimization issues of the 22 benchmark functions and the four engineering optimization design issues. The findings of the simulation on the 22 benchmark functions show that the rat optimization algorithm possesses better exploration and exploitation compared to the current rat swarm optimizer because of the random search strategy and two hunting strategies that are added to the algorithm. To enhance the global search capability of the algorithm, the Levy flight strategy is added to the rat optimization algorithm and the rat swarm optimizer, respectively, and a simulation comparison is made between the two algorithms. The comparison outcomes show that the optimization performance of the rat optimization algorithm with the Levy flight strategy is better than that of the rat swarm optimizer with the Levy flight strategy. Compared with the other well-known algorithms also shows that the novel rat optimization algorithm possesses outstanding optimization ability and faster convergence speed. Furthermore, the rat optimization algorithm performs the most outstandingly in seeking out the minimum cost in the practical engineering design issues. In addition, by comparing the optimization performance of the rat optimization algorithm with and without the Levy flight strategy in actual engineering design, it can be seen that adding some strategies to the original algorithm does not necessarily enhance the optimization ability of the algorithm. For example, it can be observed from engineering examples 2–4 that the original rat optimization algorithm proposed in this paper has better optimization ability. This also gives us a certain revelation: when we improve the algorithm, we should not blindly increase the strategy. In future work, strategies such as opposite-based learning and ranking-based mutation operators will be integrated into the proposed rat optimization algorithm to investigate whether the addition of these strategies can improve the robustness, stability, and convergence of this algorithm. In addition, the presented rat optimization algorithm will be used to solve the path-planning problem of motion agricultural robots in practical engineering.

## Figures and Tables

**Table 1:** Benchmark test functions.

Benchmark Test Functions	Dim	Range	$f_{\min}$
$f_{1}=\sum_{i=1}^{n}x_{i}^{2}$	30	[−100, 100]	0
$f_{2}(x)=\sum_{i=1}^{n}\left|x_{i}\right|+\prod_{i=1}^{n}\left|x_{i}\right|$	30	[−10, 10]	0
$f_{3}(x)=\sum_{i=1}^{n}{\left(\sum_{j=1}^{i}x_{j}\right)}^{2}$	30	[−100, 100]	0
$f_{4}(x)=\max_{i}\{|x_{i}|,1\leq i\leq n\}$	30	[−100, 100]	0
$f_{5}(x)=\sum_{i=1}^{n-1}\left[100{\left(x_{i+1}-x_{i}^{2}\right)}^{2}+{\left(x_{i}-1\right)}^{2}\right]$	30	[−30, 30]	0
$f_{6}(x)=\sum_{i=1}^{n}{\left([x_{i}+0.5]\right)}^{2}$	30	[−100, 100]	0
$f_{7}(x)=\sum_{i=1}^{n}ix_{i}^{4}+random[0,1)$	30	[−1.28, 1.28]	0
$f_{8}(x)=\sum_{i=1}^{n}\left(-x_{i}\sin(\sqrt{\left|x_{i}\right|}\right))$	30	[−500,500]	−12,569
$f_{9}(x)=\sum_{i=1}^{n}\left[x_{i}^{2}-10\cos(2\pi x_{i})+10\right]$	30	[−5.12, 5.12]	0
$\begin{array}{l} f_{10}(x)=-20\exp\left(-0.2\sqrt{\frac{1}{n}\sum_{i=1}^{n}x_{i}^{2}}-\exp\left(\frac{1}{n}\sum_{i=1}^{n}\cos2\pi x_{i}\right)\right)\\ +20+e \end{array}$	30	[−32, 32]	0
$f_{11}(x)=\frac{1}{4000}\sum_{i=1}^{n}\left(x_{i}^{2}\right)-\prod_{i=1}^{n}\cos\left(\frac{x_{i}}{\sqrt{i}}\right)+1$	30	[−600, 600]	0
$\begin{array}{l} f_{12}(x)=\frac{\pi }{n}\left\{10\sin^{2}(\pi y_{1})+\sum_{i=1}^{n-1}\begin{array}{l} {\left(y-1\right)}^{2}[1+10\sin^{2}(\pi y_{1})]\\ +{\left(y_{n}-1\right)}^{2} \end{array}\right\}\\ +\sum_{i=1}^{n}u(x_{i},10,100,4)\\ y_{i}=1+\frac{x_{i}+1}{4}\\ u(x_{i},a,k,m)=\left\{ \begin{array}{l} k{\left(x_{i}-a\right)}^{m},x^{i}>a\\ 0,-a\leq x_{i}\leq a\\ k{\left(-x_{i}-z\right)}^{m},x_{i}<a \end{array}\right. \end{array}$	30	[−50, 50]	0
$\begin{array}{l} f_{13}(x)=0.1\left\{\sin^{2}\left(3\pi x_{1}+\sum_{i=1}^{n}\begin{array}{l} {\left(x_{i}-1\right)}^{2}[1+\sin^{2}(3\pi x_{i}+1)]\\ +{\left(x_{n}-1\right)}^{2}[1+\sin^{2}(2\pi x_{n})] \end{array}\right)\right\}\\ +\sum_{i=1}^{n}u(x_{i},5,100,4) \end{array}$	30	[−50, 50]	0
$f_{14}(x)=\sum_{i=1}^{11}{\left[a_{i}-\frac{x_{1}(b_{i}^{2}+b_{i}x_{2})}{b_{i}^{2}+b_{i}x_{3}+x_{4}}\right]}^{2}$	4	[−5, 5]	0.000307
$f_{15}(x)=4x_{1}^{2}-2.1x_{1}^{4}+\frac{1}{3}x_{1}^{6}+x_{1}x_{2}-4x_{2}^{2}+4x_{2}^{4}$	2	[−5, 5]	−1.0316
$f_{16}(x)=-\frac{1+\cos(12\sqrt{x_{1}^{2}+x_{2}^{2}})}{0.5(x_{1}^{2}+x_{2}^{2})+2}$	2	[−5.12, 5.12]	−1
$f_{17}(x)=-\sum_{i=1}^{5}{\left[(x-a_{i}){\left(x-a_{i}\right)}^{T}+c_{i}\right]}^{-1}$	4	[0, 10]	−10.1532
$f_{18}(x)=-\sum_{i=1}^{7}{\left[(x-a_{i}){\left(x-a_{i}\right)}^{T}+c_{i}\right]}^{-1}$	4	[0, 10]	−10.4029
$f_{19}(x)=-\sum_{i=1}^{10}{\left[(x-a_{i}){\left(x-a_{i}\right)}^{T}+c_{i}\right]}^{-1}$	4	[0, 10]	−10.5364
$f_{20}(x)=-\cos(x_{1})\cos(x_{2})\exp(-{\left(x_{1}-\pi \right)}^{2}-{\left(x_{2}-\pi \right)}^{2})$	2	[−2π,2π]	−1
$f_{21}(x)=0.5+\frac{\sin^{2}\left(\sqrt{x_{1}^{2}+x_{2}^{2}}\right)-0.5}{{\left(1+0.001(x_{1}^{2}+x_{2}^{2})\right)}^{2}}$	2	[−100,100]	−1
$f_{22}(x)=\sum_{i=1}^{n}x_{i}\sin(x_{i})+0.1x_{i}$	10	[−10,10]	0

**Table 2:** Parameter settings for all algorithms.

Algorithm	Parameters	Values
LRSO [24]	Control parameter *R* Constant parameter *C* A power coefficient δ	[1, 5] [0, 2] (1, 3]
PSO [17]	Constant inertia ω First acceleration coefficient $c_{1}$ Second acceleration coefficient $c_{2}$	0.7298 1.4962 1.4962
ROA	Control parameter *R*	[1, 5]
	Constant parameter *C*	[0, 2]
	Random number *r*	[−2, 1]
	Constant parameter Ξ	15
	Random number q	[0, 1]
	Control parameter *A*	[0, 5]
WHO [19]	Crossover percentage PC	0.13
	Stallions percentage (number of groups) PS	0.2
	Crossover	Mean
RSO [7]	Control parameter *R*	[1, 5]
	Constant parameter *C*	[0, 2]
WOA [18]	Random number $r_{1}$	[0, 1]
	Random number $r_{2}$	[0, 1]
	Convergence factor α	[0, 2]
	Constant coefficient b	1
	Random number l	[−1, 1]
IROA	Control parameter *R*	[1, 5]
	Constant parameter *C*	[0, 2]
	Random number *r*	[−2, 1]
	Constant parameter Ξ	15
	Random number q	[0, 1]
	Control parameter *A*	[0, 5]
	A power coefficient del	(1, 3]

**Table 3:** Simulation outcomes for unimodal functions.

Function	Result	LRSO	PSO	ROA	WHO	RSO	WOA	IROA
$f_{1}$	Best	0	6.97951E−20	0	2.905E−118	0	4.3095E−179	0
	Worst	0	8.22635E−17	0	1.2353E−106	0	6.7023E−163	0
	Mean	0	7.0775E−18	0	7.6554E−108	0	3.3609E−164	0
	Std	0	1.83246E−17	0	2.7806E−107	0	0	0
$f_{2}$	Best	0	1.00253E−11	0	5.76891E−67	0	1.3735E−108	0
	Worst	9.6209E−234	1.00063E−06	1.3868E−222	2.30454E−58	1.9595E−280	3.17278E−97	0
	Mean	4.8253E−235	7.42274E−08	7.2713E−224	3.23975E−59	9.8209E−282	2.80435E−98	0
	Std	0	2.29119E−07	0	6.65451E−59	0	8.64816E−98	0
$f_{3}$	Best	0	3.879473	0	2.754E−74	0	2592.391946	0
	Worst	0	78.357014	9.1477E−166	5.38063E−62	0	28090.27522	0
	Mean	0	24.579598	4.5738E−167	2.72677E−63	0	9217.009615	0
	Std	0	21.229234	0	1.20234E−62	0	6234.494227	0
$f_{4}$	Best	0	0.479590	0	2.05305E−44	0	3.69191E−07	0
	Worst	1.8073E−233	1.738073	7.9653E−212	4.97836E−40	2.0589E−231	76.496568	0
	Mean	9.2219E−235	0.980672	3.9827E−213	7.03407E−41	1.0296E−232	19.652248	0
	Std	0	0.374931	0	1.3221E−40	0	27.141143	0
$f_{5}$	Best	28.087176	13.584130	0.014849	23.106312	28.104243	26.270804	0.247444
	Worst	28.980220	97.432220	28.809266	24.719644	28.974228	27.843659	28.703251
	Mean	28.744487	43.812144	25.838063	24.167082	28.667839	26.783466	22.632876
	Std	0.293042	32.169485	8.817368	0.446709	0.300577	0.425789	10.672547
$f_{6}$	Best	1.752414	5.10634E−20	0.225324	8.62099E−19	2.306565	0.001563	0.001792
	Worst	4.028140	7.01125E−18	2.010533	3.30477E−13	3.982083	0.009213	1.175447
	Mean	2.844619	1.89833E−18	0.737547	1.88264E−14	3.109607	0.004359	0.182260
	Std	0.715023	2.17589E−18	0.499412	7.37735E−14	0.501211	0.002179	0.280068
$f_{7}$	Best	1.10647E−06	0.183991	8.53246E−07	2.45282E−05	2.95561E−06	2.84523E−05	4.50183E−07
	Worst	0.000171	1.240802	0.000140	0.000825	0.000380	0.003421	0.000155
	Mean	4.96768E−05	0.696282	5.27615E−05	0.000383	0.000121	0.000819	3.25509E−05
	Std	4.30204E−05	0.349530	4.10437E−05	0.000217	0.000106	0.000854	3.54351E−05

**Table 4:** Simulation outcomes for multimodal functions.

Function	Result	LRSO	PSO	ROA	WHO	RSO	WOA	IROA
$f_{8}$	Best	−10612.38893	−1.7947E+308	−12569.48656	−10092.43082	−7038.363268	−12569.10738	−12569.47138
	Worst	−5374.094238	−1.6253E+308	−11979.5788	−8049.294009	−3419.628051	−8513.041244	−12568.55747
	Mean	−7198.80449	65535	−12538.44059	−9221.268766	−5544.580839	−11640.30301	−12569.19065
	Std	1361.198213	65535	131.568977	580.156324	1166.28717	1303.441748	0.296197
$f_{9}$	Best	0	38.803332	0	0	0	0	0
	Worst	0	95.515746	0	0	0	5.68434E−14	0
	Mean	0	63.428491	0	0	0	2.84217E−15	0
	Std	0	14.625901	0	0	0	1.27106E−14	0
$f_{10}$	Best	4.44089E−16	1.62143E−10	4.44089E−16	4.44089E−16	4.44089E−16	4.44089E−16	4.44089E−16
	Worst	4.44089E−16	2.660504	4.44089E−16	3.9968E−15	3.9968E−15	7.54952E−15	4.44089E−16
	Mean	4.44089E−16	0.803429	4.44089E−16	1.5099E−15	9.76996E−16	3.28626E−15	4.44089E−16
	Std	0	0.793259	0	1.67035E−15	1.30153E−15	2.47216E−15	0
$f_{11}$	Best	0	0	0	0	0	0	0
	Worst	0	0.068519	0	0	0	0	0
	Mean	0	0.020725	0	0	0	0	0
	Std	0	0.021629	0	0	0	0	0
$f_{12}$	Best	0.049588	1.9135E−17	0.008726	4.62457E−21	0.193623	0.000214	5.61659E−05
	Worst	0.316243	2.190090	0.967721	0.103669	0.875380	0.076575	0.081612
	Mean	0.184648	0.473425	0.157909	0.010366	0.348704	0.004488	0.006593
	Std	0.084444	0.673559	0.274969	0.031908	0.165451	0.016975	0.018107
$f_{13}$	Best	2.702126	1.66801E−18	5.79994E−07	3.18378E−18	2.700906	0.003615	0.001358
	Worst	2.900834	0.109867	0.315601	0.098882	2.901529	0.285479	0.068371
	Mean	2.808360	0.006592	0.049237	0.020784	2.811370	0.043155	0.016788
	Std	0.061283	0.024541	0.078648	0.031852	0.049023	0.060494	0.017467

**Table 5:** Simulation outcomes for fixed-dimension multimodal functions.

Function	Result	LRSO	PSO	ROA	WHO	RSO	WOA	IROA
$f_{14}$	Best	0.00336	0.000307	0.000316	0.000307	0.000459	0.000311	0.000307
	Worst	0.00138	0.001073	0.001578	0.001488	0.001424	0.002251	0.000488
	Mean	0.000629	0.000845	0.000629	0.000549	0.000918	0.000689	0.000357
	Std	0.000262	0.000284	0.000356	0.000433	0.000318	0.000509	5.34426E−05
$f_{15}$	Best	−1.031620	−1.031628	−1.031628	−1.031628	−1.031626	−1.031628	−1.031628
	Worst	−1.031001	−1.031628	−1.031448	−1.031628	−1.031473	−1.031628	−1.031609
	Mean	−1.031535	−1.031628	−1.031616	−1.031628	−1.031591	−1.031628	−1.031624
	Std	0.000144	2.27813E−16	4.11599E−05	1.76463E−16	3.79607E−05	5.32821E−11	4.49442E−06
$f_{16}$	Best	−1	−1	−1	−1	−1	−1	−1
	Worst	−1	−1	−1	−1	−1	−0.936245	−1
	Mean	−1	−1	−1	−1	−1	−0.987249	−1
	Std	0	0	0	0	0	0.026164	0
$f_{17}$	Best	−4.985129	−10.153199	−10.153199	−10.153199	−3.824187	−10.153191	−10.151874
	Worst	−0.497261	−2.630471	−2.527743	−2.630471	−0.350654	−5.048672	−10.121451
	Mean	−2.788463	−6.272854	−8.795370	−9.030029	−1.176110	−9.896994	−10.144520
	Std	2.004038	3.386667	2.379165	2.743155	1.021707	1.141177	0.007781
$f_{18}$	Best	−5.024169	−10.402940	−10.402939	−10.402940	−3.920794	−10.402908	−10.402623
	Worst	−0.522395	−3.724300	−4.752318	−3.724300	−0.500230	−5.087590	−10.347577
	Mean	−2.394755	−8.414489	−8.787965	−9.805302	−1.159582	−10.117281	−10.389813
	Std	1.963167	2.806450	2.388104	1.853547	0.869541	1.185167	0.014235
$f_{19}$	Best	−5.046108	−10.536409	−10.536407	−10.536409	−2.664262	−10.536396	−10.534879
	Worst	−0.943033	−2.421734	−3.552736	−3.835426	−0.655090	−2.421679	−10.517874
	Mean	−3.670737	−7.340875	−8.203867	−9.330235	−1.252252	−8.913221	−10.529964
	Std	1.834910	3.687014	2.762350	2.494043	0.607943	2.962852	0.004097
$f_{20}$	Best	−0.999960	−1	−1	−1	−0.999594	−1	−0.999999
	Worst	−0.986001	−1	−0.999904	−1	−0.988724	−0.999999	−0.999994
	Mean	−0.997543	−1	−0.999993	−1	−0.996964	−0.999999	−0.999998
	Std	0.003211	0	2.14675E−05	0	0.002723	3.33419E−08	1.39629E−06
$f_{21}$	Best	−1	−1	−1	−1	−1	−1	−1
	Worst	−1	−0.990284	−1	−1	−1	−0.990284	−1
	Mean	−1	−0.999514	−1	−1	−1	−0.996113	−1
	Std	0	0.002172	0	0	0	0.004883	0
$f_{22}$	Best	0	3.0838E−33	0	5.6902E−100	0	3.3978E−111	0
	Worst	8.4841E−250	4.88498E−15	6.9799E−220	2.97059E−05	6.9287E−240	3.938420	0
	Mean	4.2421E−251	1.57374E−15	5.3043E−221	3.00607E−06	3.4644E−241	0.626839	0
	Std	0	1.42295E−15	0	7.92946E−06	0	1.257635	0

**Table 6:** Optimization comparison of the piston lever design issue.

Algorithm	Optimal Value for Variables				Optimal Cost
	H	B	X	D	
LRSO [24]	0.05	1.04322	120	2.053458	1.1373
RSO [7]	0.05	2.08281	62.8666	2.79619	4.0768
ROA	0.05	1.109675	120	2.049557	1.1982
IROA	0.05	1.025158	120	2.050285	1.116
WOA [18]	0.05617301	1.033396	120	1.033396	1.1384
SCSO [34]	0.050	2.040	119.99	4.083	8.40901438899551
CSO [34]	0.050	2.399	85.68	4.0804	13.7094866557362
GWO [34]	0.060	2.0390	120	4.083	8.40908765909047
WAO [34]	0.099	2.057	118.4	4.112	9.05943208079399
SSA [34]	0.050	2.073	116.32	4.145	8.80243253777633
GSA [34]	497.49	500	60.041	2.215	168.094363238712
BWO [34]	12.364	12.801	172.02	3.074	95.9980864948937
PDO [35]	0.05	0.144897318	120	4.11572157	4.602
DMOA [35]	0.05	0.125073578	120	4.116042166	4.695
AOA [35]	0.05	0.125073578	120	4.116042166	7.738
CPSOGSA [35]	500	500	120	2.578147082	4.6949
BBO [35]	129.4	2.43	119.8	4.75	4.6956
SCA [35]	0.05	0.144897318	120	4.11572157	4.6977
ISA [35]	N/A	N/A	N/A	N/A	8.4
CGO [35]	N/A	N/A	N/A	N/A	8.41281381
MGA [35]	N/A	N/A	N/A	N/A	8.41340665

**Table 7:** Optimization comparison of the welded beam design issue.

Algorithm	Optimal Value for Variables				Optimal Cost
	h	l	t	b	
LRSO [24]	0.17092	4.1264	9.0754	0.20679	1.7698
RSO [7]	0.25105	4.4848	6.0690	0.75568	4.1005
ROA	0.20496	3.2877	9.0361	0.20575	1.6989
IROA	0.19181	3.5215	9.0373	0.20573	1.7104
WOA [18]	0.18657	3.6653	9.0742	0.20554	1.7261
BBO [36]	0.1854860	4.3129000	8.4399030	0.2359020	1.9180550
PSO [36]	0.219292	3.430416	8.433559	0.236204	1.852720
ICA [36]	0.205799	3.469634	9.034950	0.205806	1.725135
WSA [36]	0.20573000	3.47048900	9.03662400	0.20573000	1.72485200
CBO [36]	0.2057220	3.4704100	9.0372760	0.2057350	1.7246630
GWO [36]	0.2056770	3.4708940	9.0385580	0.2057390	1.7252320
CMA-ES [37]	0.5617	4.3786	4.6772	0.9286	2.28384
L-SHADE [37]	0.4819	3.2140	5.4763	0.5753	3.43372
EHO [37]	1.0149	4.7616	4.8130	0.8722	3.36770
GOA [37]	0.4069	2.1411	6.3834	0.4123	2.43534
HHO [37]	0.1961	3.7449	9.0061	0.2071	1.75163
HBA [37]	0.2057	3.4704	9.0366	0.2057	1.72451
TSA [38]	0.205563	3.474846	9.035799	0.205811	1.725661
TLBO [38]	0.204695	3.536291	9.004290	0.210025	1.759173
NGO [38]	0.20576	2.471	9.0361	0.20577	1.725202

**Table 8:** Optimization comparison of the gear train design issue.

Algorithm	Optimal Value for Variables				Optimal Cost
	$N_{A}$	$N_{B}$	$N_{C}$	$N_{D}$	
LRSO [24]	16.6343	12	12	60	7.8647E−13
RSO [7]	18.4336	12	12	39.4764	0.0028737
ROA	58.7768	25.7646	17.2843	52.513	0
IROA	28.9615	13.4345	17.8644	57.4365	9.257E−21
WOA [18]	32.9552	12.8789	13.3838	36.252	0
GJO [39]	56.7899	18.5239	19.8299	44.8316	1.7754E−19
GTO [40]	34.65788	12	12	28.79761	2.42E−18
DE [40]	31.06397	12	12.01193	32.16129	9.75E−10
GSA [40]	52.08397	18.17396	21.96011	52.70814	2.70E−12
WOA [40]	42.65045	15.93899	18.69951	49.38557	2.70E−12
KABC [41]	50.4259	22.3987	16.7082	51.4394	0

**Table 9:** Optimization comparison of the car side impact design issue.

Algorithm	Optimal Value for Variables						Optimal Cost
	$x_{1}$	$x_{2}$	$x_{3}$	$x_{4}$	$x_{5}$	$x_{6}$	
	$x_{7}$	$x_{8}$	$x_{9}$	$x_{10}$	$x_{11}$		
LRSO [24]	0.5	0.824286	0.5	1.44438	0.5	1.49778	
	0.5	1	0.0146386	−19.4479	−4.88457		21.465
RSO [7]	0.5	1.03301	0.5	1.28331	0.501519	0.536513	
	0.5	0.17284	0.24126	3.17658	16.4998		22.2139
ROA	0.5	0.972586	0.5	1.02759	0.5	0.5	
	0.5	0.850574	0.513587	26.8917	22.6441		20.7828
IROA	0.5	0.952691	0.5	1.07141	0.5	0.5	
	0.5	0.644747	0.262471	21.976	21.8061		20.8258
WOA [18]	0.5	0.958512	0.5	1.18049	0.5	0.5	
	0.5	0.786177	0.0193357	21.623	−30		21.302
DE [42]	0.5	1.1167	0.5	1.30208	0.5	1.5	
	0.5	0.345	0.192	−19.54935	−0.00431		22.84474
FA [42]	0.5	1.36	0.5	1.202	0.5	1.12	
	0.5	0.345	0.192	8.87307	−18.99808		22.84298
TLBO [42]	0.5	1.1135	0.5	1.307	0.5	1.5	
	0.5	0.345	0.192	−20.0655	0.1139		22.8436
TLCS [42]	0.5	1.1163	0.5	1.3023	0.5	1.5	
	0.5	0.345	0.192	−19.5721	0.0157		22.8430
CPA [42]	0.5	1.1157586	0.5	1.30321196	0.5	1.5	
	0.5	0.345	0.27247957	−19.67009727	0.00000206		22.84298982
ABC [43]	0.5	1.0624205	0.5148211	1.4491503	0.5	1.5	
	0.5	0.345	0.192	−29.34755	0.7410998		23.17588963
MFO [43]	0.5	1.116539	0.5	1.301908	0.5	1.5	
	0.5	0.345	0.345	−19.5304	−0.000006		22.84297087
ALO [43]	0.5	1.11596	0.5	1.30286	0.5	1.5	
	0.5	0.345	0.192	−19.6330	0.023649		22.84298071
ER−WCA [43]	0.5	1.118688	0.5	1.298407	0.5	1.5	
	0.5	0.345	0.192	−19.1461	−0.01527		22.84326462
GWO [43]	0.5	1.111484	0.5	1.312203	0.501214	1.5	
	0.5	0.345	0.192	−20.6057	−25531		22.85279276
WCA [43]	0.5	1.1155932	0.5	1.3034919	0.5000146	1.5	
	0.5	0.345	0.192	−19.69967	−0.023854		22.84303648
MBA [43]	0.5	1.1172701	0.5	1.30008438	0.5	1.499987	
	0.5	0.345	0.345	−19.40045	−0.379205		22.84359640
SSA [43]	0.5	1.1093195	0.5	1.3148	0.5	1.499999	
	0.5	0.345	0.192	−20.821793	0.4412962		22.84651410
WOA [43]	0.5	1.108001	0.534477	1.30577	0.5	1.473844	
	0.5	0.345	0.192	−19.69924	3.4816923		23.04216220
CSS [43]	0.5	1.184389	0.5	1.230036	0.5	1.5	
	0.5	0.280792	0.342425	−7.394733	0.042206		23.00733588

## Data Availability

The data presented in this study are available on request from the corresponding author.
